# Bias and precision of SPECT-based ^177^Lu activity-concentration estimation using a ring-configured solid-state versus a dual-headed anger system

**DOI:** 10.1186/s40658-024-00693-5

**Published:** 2024-11-04

**Authors:** Anna Stenvall, Irma Ceric Andelius, Elias Nilsson, Albin Lindvall, Erik Larsson, Johan Gustafsson

**Affiliations:** 1 Radiation Physics, Department of Haematology, Oncology, and Radiation Physics, Skåne University Hospital, Lund, Sweden; 2 Department of Translational Medicine and Wallenberg Centre of Molecular Medicine, Lund University, Malmö, Sweden; 3 Department of Nuclear Medicine and Medical Physics, Karolinska University Hospital, Stockholm, Sweden; 4 Medical Radiation Physics, Lund, Lund University, Lund, Sweden

**Keywords:** Quantitative SPECT, Lutetium-177, Ring-configured SPECT, Cadmium-zinc-telluride

## Abstract

**Background:**

The aim was to compare bias and precision for ^177^Lu-SPECT activity-concentration estimation using a dual-headed Anger SPECT system and a ring-configured CZT SPECT system. This was investigated for imaging at 208 keV and 113 keV, respectively.

**Methods:**

Phantom experiments were performed on a GE Discovery 670 system with 5/8’’ NaI(Tl) crystal (dual-headed Anger system) and a GE StarGuide (ring-configured CZT system). Six spheres (1.2 mL to 113 mL) in a NEMA PET body phantom were filled with ^99m^Tc and ^177^Lu, separately. Mean relative errors and coefficients of variation (CV) in estimated sphere activity concentration were studied over six timeframes of 10 min each for the two systems. For ^177^Lu, similar acquisitions were also performed for an anthropomorphic phantom with two spheres (10 mL and 25 mL) in a liver with non-radioactive background and a sphere-to-background ratio of 15:1. Tomographic reconstruction was performed using OS-EM with 10 subsets with compensation for attenuation, scatter, and distance-dependent spatial resolution. For the Anger system, up to 40 iterations were used and for the ring-configured CZT system up to 30 iterations were used.

**Results:**

The two systems showed similar mean relative errors and CVs for ^177^Lu when using an energy window around 208 keV, while the ring-configured system demonstrated a lower bias for a similar CV compared to the Anger system for ^99m^Tc and for ^177^Lu when using an energy window around 113 keV. However, total activity in the phantom tended to be overestimated in both systems for these cases.

**Conclusions:**

The ring-configured CZT system is a viable alternative to the dual-headed Anger system equipped with medium-energy collimators for ^177^Lu-SPECT and shows a potential advantage for activity-concentration estimation when operated at 113 keV. However, further consideration of the preservation of total activity is warranted.

## Background

Quantitative single photon emission computed tomography (SPECT) is the basis for image-based dosimetry in radionuclide therapy (RNT), and as several new forms of RNT have been introduced in recent years, interest in imaging of patients undergoing therapy has also increased [1, 2]. One of the most important radionuclides for contemporary RNT is ^177^Lu for which quantitative SPECT and dosimetry have been described in international guidelines [3, 4].

The major hardware development for gamma cameras that has reached commercial systems during last years is the use of the semi-conductor cadmium-zinc-telluride (CZT) as detector material [5, 6], which has several advantages compared with the old design of a scintillator material (typically NaI(Tl)) combined with Anger logic [7]. In addition to the better energy resolution of CZT compared with NaI(Tl), the introduction of semiconductors in SPECT imaging has allowed for the development of new system designs with better spatial resolution and sensitivity than standard dual-headed systems with parallel-hole collimators [8]. One such example is systems with a ring of radially movable and transaxially sweeping detectors, commercialized as the Veriton™ (Spectrum Dynamics, Caesarea, Israel) and StarGuide™ (GE HealthCare, Haifa, Israel) systems. Such systems have been demonstrated to be advantageous for several diagnostic tasks in nuclear medicine [9–11].

While primarily designed for diagnostic imaging, CZT-based gamma-cameras and ring-configured SPECT systems have also been explored for imaging of therapeutic compounds, *e.g.*, ^177^Lu-labelled radiopharmaceuticals [12–15]. The increased sensitivity of ring-configured systems compared with dual-headed cameras has been argued to allow for substantially shortened acquisition times [16, 17], which is useful for the clinical implementation of dosimetry-guided RNT. However, there are also a number of issues related to CZT detectors that are potentially problematic for their use in quantitative SPECT. Firstly, the design with pixelated detectors makes collimator alignment important [18, 19] and collimators are typically fixed on current systems. The focus on ^99m^Tc in system designs makes these collimators sub-optimal for imaging of medium- and high-energy photons, such as the 208 keV photons emitted in the decay of ^177^Lu, due to septal penetration. Some studies have therefore focused on imaging of the 113 keV from ^177^Lu and then obtained reasonable accuracy in estimated activity [13–15], but there is also an interest in using the information from a 208 keV energy window [12]. Secondly, pixelated CZT detectors suffer from the charge-sharing phenomenon, leading to a low-energy tail of primary photons in acquired spectra, which in turn leads to complications for window-based scatter compensation techniques [20, 21].

Ring-configured SPECT systems have been thoroughly characterized and compared with conventional dual-headed Anger systems for diagnostic imaging [9, 11, 22, 23]. However, improved image quality for diagnostic tasks is not necessarily transferable to quantification [24], which is the relevant task for RNT dosimetry. One of the main arguments for the use of ring-configured systems instead of conventional Anger systems for quantitative ^177^Lu SPECT has been to shorten acquisition times [14, 17]. However, when comparing SPECT for a Siemens Anger system using the 208 keV photon emission of ^177^Lu and SPECT with a Veriton ring configured CZT system using the 113 keV photon emission, Nuttens et al. [15] found the ring-configured system to result in a lower contrast than the Anger system for similar background variabilities for phantom measurements. Furthermore, comparing noise versus bias properties between systems is often difficult as it depends on the camera specifications (detector material, collimator design, etc.) and acquisition settings (*e.g.*, energy window settings and acquisition time) as well as reconstruction and post-processing parameters. The possibility of using ring-configured CZT systems for ^177^Lu activity-concentration estimation is well established [12, 14, 15], but the possible advantage of these systems for this task compared with conventional Anger systems is still unclear and further research is warranted.

The aim of this paper is to experimentally evaluate a ring-configured CZT gamma-camera system (GE StarGuide, GE HealthCare) for ^177^Lu activity-concentration estimation and compare it with a conventional dual-headed Anger system (GE Discovery 670, GE HealthCare) with respect to systematic and random errors. This is performed using phantom measurements for ^99m^Tc and the separate peaks of ^177^Lu (208 keV and 113 keV), where the former radionuclide is used to establish a baseline for comparison with the latter.

## Methods

### SPECT systems

Two SPECT/CT-systems were evaluated, one conventional dual-headed Anger system (GE Discovery 670, GE HealthCare) with a 5/8″ NaI(Tl) crystal, equipped with low-energy high-resolution (LEHR) or medium-energy general-purpose (MEGP) collimators, and a ring-configured CZT gamma-camera system (GE StarGuide, GE HealthCare). Both systems are installed at Skåne University Hospital in Lund.

The StarGuide SPECT/CT-system is a ring-configured CZT SPECT system with 12 detector units installed within a circular gantry. Each detector unit consists of a column of seven CZT modules, forming an axial field of view (FOV) of 27.5 cm. Each of the seven CZT modules is composed of a 7.25 mm CZT crystal with 16 × 16 anode elements, with an anode-element pitch of 2.46 × 2.46 cm^2^, and a non-removable tungsten collimator adapted to the element grid. The energy range of the CZT detectors on the StarGuide system is 40 keV to 279 keV. The system can be run in low energy mode (40 keV to 200 keV) or in medium energy mode, with a coarser sampling of the energy spectrum, if acquiring projections for photons with energies over 200 keV, *i.e.* enabling acquisition of both energy peaks of ^177^Lu (113 keV and 208 keV).

### Phantom preparation

Activity meters used for determination of activities (Comecer Vik-202 and Capintec CRC-55tR) were calibrated with traceability to primary standard for ^99m^Tc and ^177^Lu.

#### Calibration and NEMA phantoms

Two different phantom geometries were utilized in order to compare the quantitative performance of ^99m^Tc and ^177^Lu for the two camera systems: *a)* a uniform cylindrical phantom (SUV phantom) and *b)* an image quality phantom (NEMA IEC Body phantom), modified to include a sphere of 113 mL instead of the smallest sphere. The spheres were positioned alternating between large and small, rather than arranged in ascending order of size, with the smallest sphere situated between the largest and the next largest spheres, and the background was filled with non-radioactive water. For each nuclide (^99m^Tc and ^177^Lu) both phantoms (*a* and *b*) were filled from the same bulk solution, having a well-defined activity concentration of either ^99m^Tc-pertechnetat or [^177^Lu]Lu-DOTA-TOC. For the ^177^Lu-bulk solution, ethylenediaminetetraacetic acid (EDTA) was added in order to bind potentially unbound ^177^Lu. The activity in each compartment (uniform cylinder and NEMA spheres) were determined by weighing each compartment pre- and post-filling. Specifications for the uniform phantom and NEMA phantom are shown in Table 1.

**Table 1:** Specifications of the phantoms prepared. Activity concentrations are specified at the time of acquisition at the Anger system

	Uniform phantom	NEMA phantom	Anthropomorphic phantom
Diameter (mm)/Volume (mL) (fillable containers inner dimensions)	- /5640	Sphere 1: 13/1.2	Sphere 1: 27/9.9
		Sphere 2: 17/2.6	Sphere 2: 36/25.3
		Sphere 3: 22/5.6	
			Liver (excluding spheres): 1724 mL
		Sphere 4: 28/11.5	
		Sphere 5: 37/26.5	
		Sphere 6: 60/113.1	
^99m^Tc			
Activity concentration (MBq/mL)	0.099	0.50	-*
^177^Lu			
Activity concentration (MBq/mL)	0.098	1.8	Spheres: 1.93 (cold)
			1.87 (warm)
			Liver: 0.13

^*^The anthropomorphic phantom was not imaged with ^99m^Tc

#### Anthropomorphic phantom

For ^177^Lu, an anthropomorphic phantom containing a liver insert with two sphere inserts inside the liver (LK-S Kyoto Liver/Kidney Phantom, Kyoto Kagaku Co., Ltd) was also used. The phantom was imaged with both cold (*i.e*., non-radioactive) and warm (*i.e.* radioactive) liver background. The liver and sphere compartments were filled with [^177^Lu]Lu-DOTA-TOC from the same bulk solution as for the NEMA phantom. The activity in each compartment was determined by weighing the spheres and the liver compartment on a weighing scale pre- and post-filling. The sphere activity concentration and the tumour-to-liver ratio (set to 15) were based on the activity concentrations for tumours and liver 24 h post administration of [^177^Lu]Lu-DOTA-TATE, determined from the pharmacokinetic model developed by Brolin et al. [25]. Phantom specifications for the anthropomorphic phantom are shown in Table 1.

### Image acquisition

#### Dual-headed Anger gamma-camera system

Projections of the NEMA phantom filled with ^99m^Tc were acquired for 120 angles in step-and-shoot mode with 60 s per projection using LEHR collimators. Data were saved in list-mode format for further processing off-line (non-disclosure agreement with GE HealthCare). Six projection datasets were created from consecutive time-intervals with 10 s per projection (*i.e.*, a measurement time of 10 min). Further acquisition parameters are specified in Table 2. Likewise, projections were acquired for the uniform cylindrical phantom using the same parameters, but the full acquisition time was used without any division into time frames. In addition, low-dose CT images (tube voltage 120 kV) were acquired for attenuation compensation.

**Table 2:** Acquisition and reconstruction parameters for the conventional Anger and ring-configured CZT-camera

	Anger camera			Ring-configured CZT camera		
	^99m^Tc	^177^Lu		^99m^Tc	^177^Lu	
*Acquisition parameters*						
Full acquisition time	60 s (120 angles) *i.e.* 60 min	120 s (60 angles) *i.e.* 60 min		60 min (decay corrected to Anger camera)		
Time per timeframe	10 s (120 angles) *i.e.* 10 min	20 s (60 angles) *i.e.* 10 min		10 min (decay corrected to Anger camera)		
Main window/keV	140.5 ± 10%	113 ± 10%	208 ± 7.5%	140.5 ± 10%	113 ± 10%	208 ± 6%
Scatter window/keV	120.1 ± 5.3%	98.9 ± 2.9%	184.6 ± 4.2%	120 ± 5%	129.4 -3.3% / + 3.68%	185 ± 5%
		127.1 ± 2.2%				
					96.6 -4.61% / + 4.43%	
Matrix size	128 × 128			16 × 112		
Pixel size/mm^2^	4.4 × 4.4			2.46 × 2.46		
*Reconstruction parameters*						
Number of iterations	1–40 (in steps of 1)			2, 5, 10, 20 and 30		
Number of Subsets	10			10		
Voxel size/mm^3^	4.4 × 4.4 × 4.4			2.46 × 2.46 × 2.46		
Scatter-compensation method	ESSE	ESSE	ESSE	DEW	TEW	DEW
	DEW	TEW	DEW			

Projections of the NEMA phantom and anthropomorphic phantom filled with ^177^Lu were acquired similarly as for the ^99m^Tc acquisition but using 60 angles with 120 s per projection and MEGP collimators. Six projection datasets with 20 s per projection (*i.e.*, a measurement time of 10 min) were created from consecutive timeframes with main energy and scatter windows as specified in Table 2. The energy windows for the ring-configured CZT system were per the manufacturer’s recommendation. The main energy windows for ^177^Lu at the Anger camera were set according to clinical routine at our institution with scatter windows set to half the width of the main window, and scatter estimates calculated according to Ogawa et al. [26] with the upper window set to zero for the DEW case. Projections were acquired for the uniform phantom using the same parameters, but the full acquisition time was used without any division into time frames.

#### Ring-configured CZT-system

The StarGuide systems requires uniformity maps to be acquired for each energy window employed. For ^99m^Tc, a uniformity map was created with the default ^57^Co line source. Before the ^99m^Tc phantom experiments, a daily quality control (QC) for low energy (LE) was performed, and before the ^177^Lu phantom experiments, the additional medium energy (ME) mode QC was performed. Uniformity maps for ^177^Lu were created using a glass pipet with approximately 2 GBq uniform activity solution, positioned in isocentre and covering the entire axial FOV.

The number of projections were determined by the system, based on the optical surface scanning. Acquisition was performed with the detectors in continuous sweep mode, and 4 or 5 gantry rotations. Data were collected for 1 h in list-mode and further reframed to six projection datasets of nominally 10 min each. The detector sweeping results in acquisition times not being arbitrarily selectable, and real acquisition times differed slightly from this nominal value. Acquisition times were also adjusted to account for physical decay compared with the time of measurement on the Anger system. Accounting for this decay, the effective acquisition times differed less than 5% between systems for ^99m^Tc and less than 1% for ^177^Lu. Main energy and scatter windows are presented in Table 2. In addition, a low dose CT scan (tube voltage 120 kV) was acquired for localization and attenuation compensation.

### SPECT reconstruction

Tomographic images for the Anger camera were reconstructed with an off-line program using OS-EM with one to 40 iterations (10 subsets) with compensation for attenuation, scatter, and distance-dependent resolution. Two sets of images were reconstructed for each main energy window. One employed model-based scatter compensation using the ESSE method [27] and one used window-based scatter compensation using the dual-energy window (DEW) (^99m^Tc 140 keV, ^177^Lu 208 keV) or triple-energy window (TEW) (^177^Lu 113 keV) methods [26, 28]. Further details are presented in Table 2.

For the ring-configured CZT system, tomographic images were reconstructed on the Smart Console version 1.6.0 (GE Healthcare) using OS-EM with 2, 5, 10, 20, and 30 iterations (10 subsets), to a voxel size of 2.46 × 2.46 × 2.46 mm^3^, with compensation for attenuation, scatter (TEW for the 113 keV energy window, modified DEW scatter compensation applied for the 208 keV window to account for primary photons in the scatter window), and distance-dependent resolution which for 208 keV also included compensation for penetration [29].

#### Calibration

Large cylindrical volumes of interest (VOIs, radius 7 cm and depth 20 cm) were placed centrally in the reconstructed images of the uniform phantom and the total signal rate in the VOI related to the product of the activity concentration determined from phantom preparation and the VOI volume, generating a calibration factor in signal rate per activity. Images for the other phantoms were divided by the calibration factor determined for the uniform phantom with the corresponding acquisition and reconstruction settings (energy window, scatter-compensation method, and number of iterations).

### Evaluation

Volumes-of-interests were defined as voxelized spheres with the same volumes as the physical spheres. The apparent mean activity concentration in the VOIs was compared with the activity concentration determined at phantom preparation. As a measure of bias, the mean relative error over timeframes of the activity concentration for sphere $i$ was computed as

1 $$\begin{array}{*{20}c} {\varepsilon_{i} = \frac{{\overline{C}_{i} }}{{C_{\mathrm{ref}} }} - 1} \\ \end{array}$$

where ${\overline{C}}_{i}$ is the mean apparent-activity concentration for sphere $i$, and ${C}_{\text{ref}}$ is the activity concentration at the start of image acquisition determined from the phantom preparation. As a measure of precision, the coefficient of variation (CV) for sphere $i$ was computed as

2 $$\begin{array}{*{20}c} {CV_{i} = \frac{{s_{i} }}{{\overline{C}_{i} }}} \\ \end{array}$$

where $s_{i}$ is the standard deviation in apparent activity concentration for sphere $i$ over the six timeframes.

The total activity was estimated by defining a VOI following the boundary of the phantom and compared with the activity at start of image acquisition estimated from phantom preparation. The mean relative error was studied as function of number of iterations.

## Results

### ^99m^Tc measurements

**Fig. 1:**
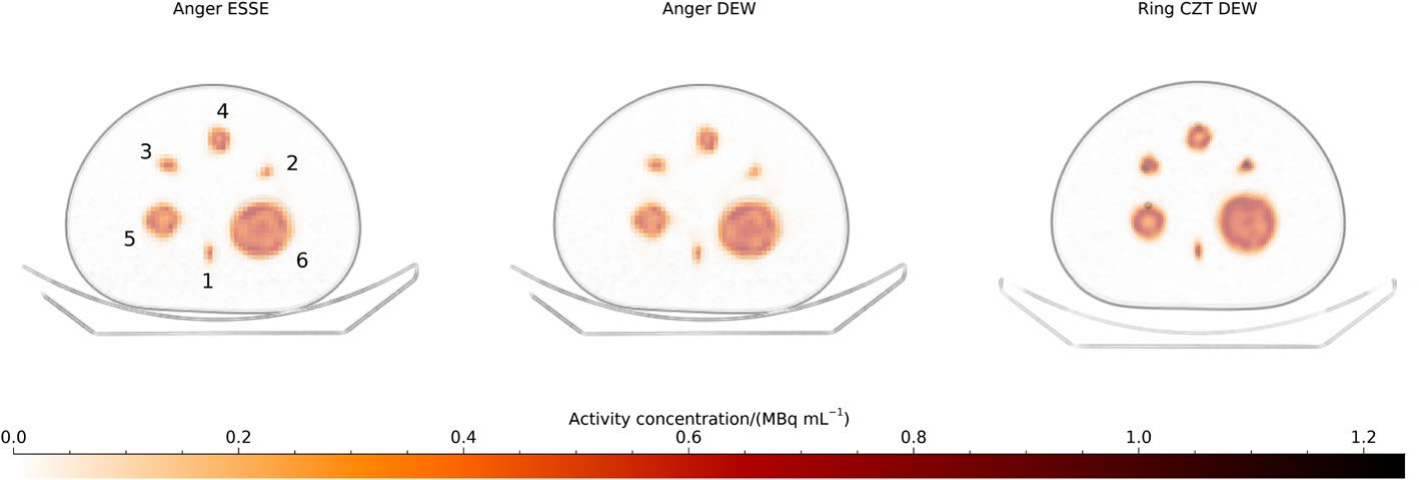
Examples of SPECT images of the NEMA phantom filled with ^99m^Tc. Images are reconstructed with 10 iterations (10 subsets). Sphere positions are indicated for the image from the Anger system using ESSE scatter compensation

Examples of transaxial SPECT images of the NEMA phantom filled with ^99m^Tc, reconstructed with 10 iterations and 10 subsets are shown in Fig. 1. The two images from the Anger system, reconstructed with different scatter-compensation methods, appear similar, whilst the SPECT image from the CZT ring-configured system appears slightly sharper. Gibbs artefacts from the resolution compensation are seen at the sphere boundaries.

**Fig. 2:**
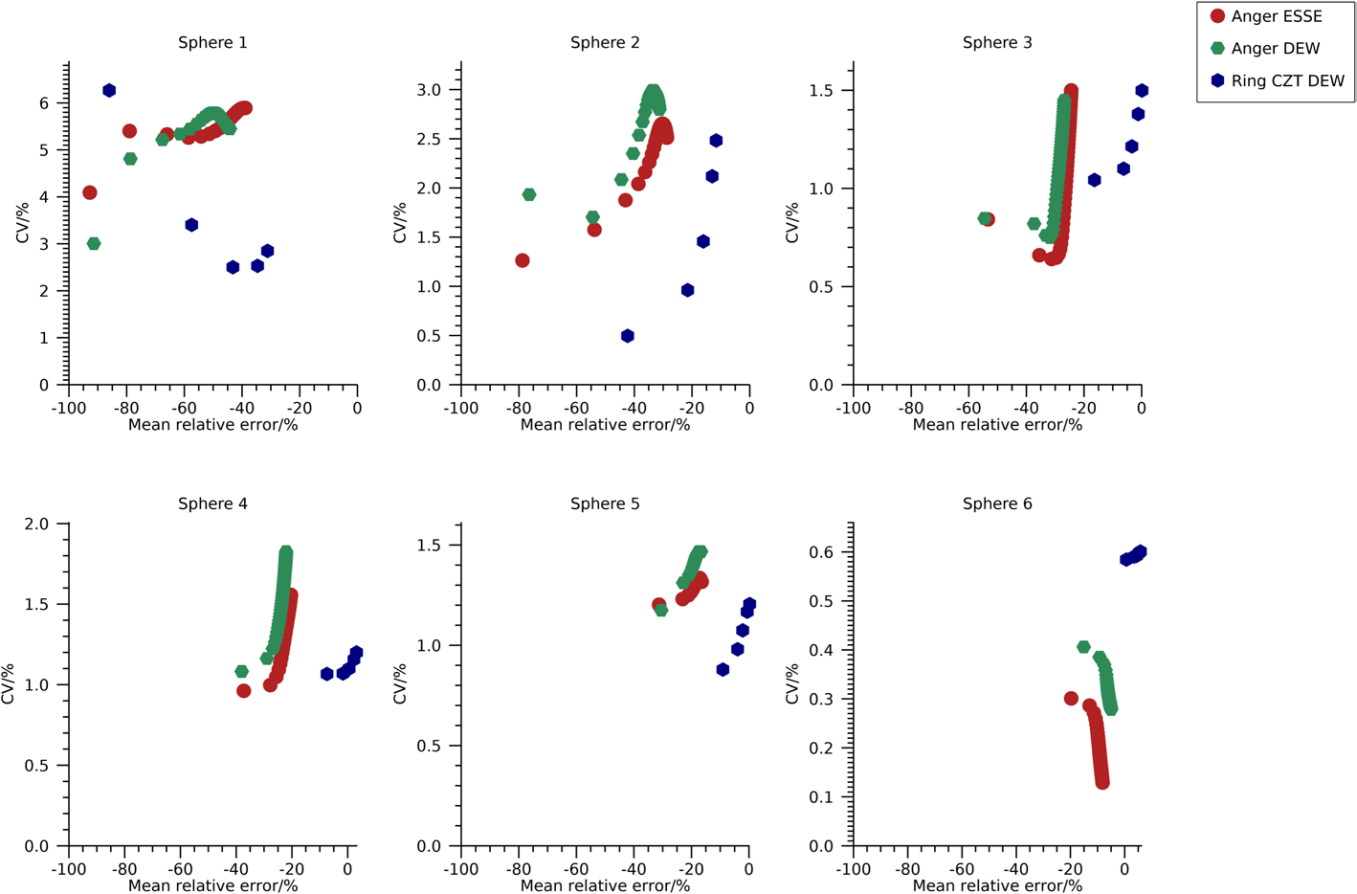
Coefficient of variation versus mean relative error for the NEMA phantom filled with ^99m^Tc. Each point represents a specific number of iterations. Note that the ordinate is individual for each sub-plot

Plots of the mean relative error versus CV over time frames are shown in Fig. 2 and numeric data at 30 iterations are presented in Table 3. The mean relative error is similar for estimates from the Anger system using the ESSE and DEW methods, but there is a tendency for slightly lower CVs for the ESSE method. The mean relative error is generally lower for estimates from the ring-configured CZT system for similar CVs as for estimates from the Anger system, in line with the visual differences in sharpness observed in Fig. 1.

**Table 3:** Summary of estimated activity concentrations at 30 iterations. Results are provided in the format mean relative error ± CV

		Sph. nr	^99m^Tc (ESSE)	^99m^Tc (DEW)		^177^Lu (ESSE 113 keV)	^177^Lu (TEW 113 keV)		^177^Lu (ESSE 208 keV)	^177^Lu (DEW 208 keV)
			Over time- frames/%	Over time- frames/%	Full time/%	Over time- frames/%	Over time- frames/%	Full time/%	Over time- frames/%	Over time- frames/%
NEMA	Anger	1	−39.9 ± 5.9	−45.4 ± 5.5	−44.7	−57.8 ± 17.5	−69.1 ± 27.5	−64.7	−62.4 ± 23.0	−65.4 ± 28.5
		2	−29.2 ± 2.6	−31.8 ± 2.9	−31.4	−43.1 ± 4.9	−51.3 ± 8.1	−48.3	−45.4 ± 2.6	−46.9 ± 2.3
		3	−25.0 ± 1.3	−27.5 ± 1.3	−27.1	−37.0 ± 2.7	−41.8 ± 4.3	−40.1	−37.9 ± 3.0	−37.8 ± 2.6
		4	−20.7 ± 1.5	−22.4 ± 1.7	−22.2	−28.5 ± 2.8	−32.7 ± 4.6	−29.9	−28.5 ± 1.7	−26.3 ± 1.6
		5	−16.8 ± 1.3	−17.0 ± 1.5	−16.7	−26.6 ± 1.0	−30.1 ± 1.7	−29.4	−26.7 ± 0.8	−26.1 ± 0.4
		6	−8.4 ± 0.1	−5.2 ± 0.3	−5.0	−19.2 ± 0.3	−19.8 ± 0.6	−19.5	−18.2 ± 0.6	−15.6 ± 0.4
	Ring CZT	1	N/A	−31.1 ± 2.8	−25.5	N/A	−47.7 ± 10.9	−40.5	N/A	−64.4 ± 16.4
		2	N/A	−11.6 ± 2.5	−9.2	N/A	−29.9 ± 3.7	−28.5	N/A	−45.1 ± 5.3
		3	N/A	0.1 ± 1.5	−1.3	N/A	−20.9 ± 3.7	−18.6	N/A	−40.1 ± 3.2
		4	N/A	3.2 ± 1.2	4.1	N/A	−12.4 ± 2.4	−10.4	N/A	−31.8 ± 1.7
		5	N/A	0.2 ± 1.2	0.4	N/A	−17.4 ± 1.6	−16.4	N/A	−33.5 ± 1.5
		6	N/A	5.8 ± 0.6	6.1	N/A	−7.1 ± 0.8	−6.2	N/A	−26.1 ± 0.6
Warm anthropomorphic	Anger	1	−*	−*	−*	−36.7 ± 2.2	−39.9 ± 2.3	−38.5	−37.0 ± 3.1	−37.1 ± 2.5
		2	−*	−*	−*	−30.0 ± 2.1	−33.1 ± 2.6	−32.3	−29.5 ± 0.8	−28.0 ± 1.2
	Ring CZT	1	−*	−*	−*	N/A	−23.1 ± 5.7	−21.7	N/A	−39.7 ± 1.6
		2	−*	−*	−*	N/A	−16.3 ± 2.6	−15.3	N/A	−32.5 ± 2.2
Cold anthropomorphic	Anger	1	−*	−*	−*	−35.3 ± 2.5	−39.8 ± 3.5	−37.6	−35.1 ± 2.4	−35.5 ± 2.6
		2	−*	−*	−*	28.6 ± 1.2	−33.2 ± 1.0	−32.7	−29.5 ± 1.1	−28.6 ± 1.2
	Ring CZT	1	−*	−*	−*	N/A	−20.5 ± 1.8	−18.7	N/A	−38.3 ± 3.2
		2	−*	−*	−*	N/A	−11.7 ± 1.9	−10.6	N/A	−31.6 ± 0.5

^*^The anthropomorphic phantom was not imaged with ^99m^Tc

**Fig. 3:**
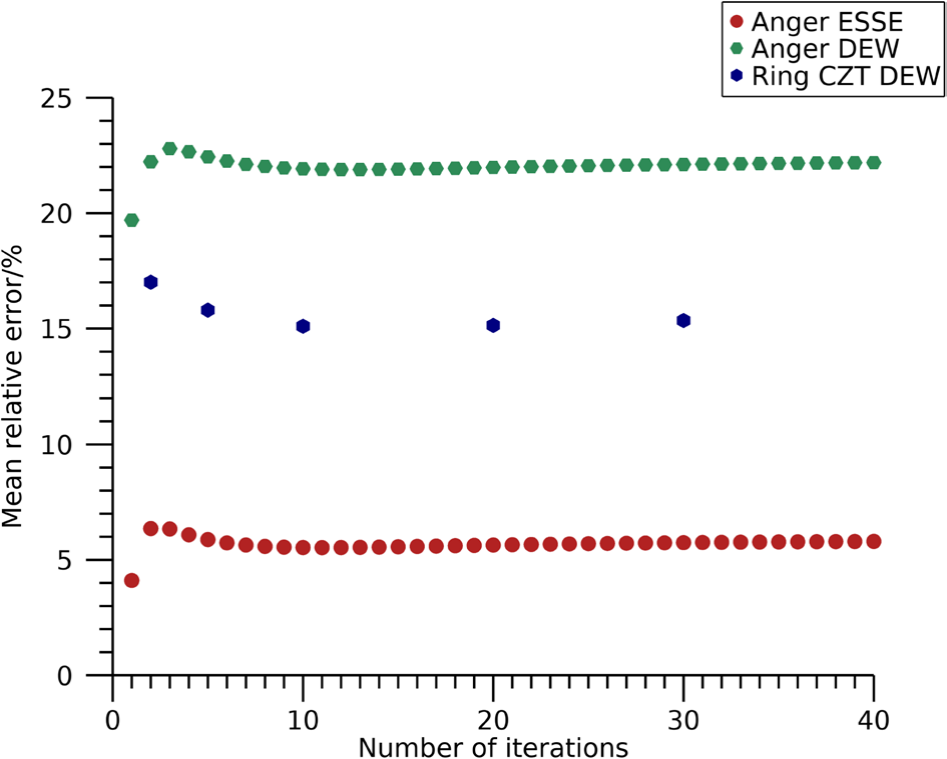
Mean relative error in estimated total activity in the NEMA phantom filled with ^99m^Tc. Results are presented as function of number of iterations. The number of updates is ten times the number of iterations

Mean relative error over time frames for total activity in the NEMA phantom are shown in Fig. 3. There is a tendency for activities to be overestimated, especially for the Anger system when using the DEW scatter-compensation method and the ring-configured CZT system. Numeric data at 30 iterations are presented in Table 4.

**Table 4:** Summary of estimated total activity in the phantom at 30 iterations. Results are provided in the format mean relative error ± CV

		^99m^Tc (ESSE)		^99m^Tc (DEW)		^177^Lu (ESSE 113 keV)		^177^Lu (TEW 113 keV)		^177^Lu (ESSE 208 keV)	^177^Lu (DEW 208 keV)
		Over time-frames/%	Over time- frames/%		Full time/%	Over time- frames/%	Over time- frames /%		Full time/%	Over time- frames/%	Over time- frames/%
NEMA	Anger	5.7	22.1		17.1	0.0	29.1		−2.1	−3.8	3.8
	Ring CZT	N/A	15.3		13.7	N/A	21.1		8.6	N/A	0.8
Warm anthropomorphic	Anger	−*	−*		−*	−1.2	24.8		3.2	−5.6	−2.1
	Ring CZT	−*	−*		−*	N/A	19.5		3.4	N/A	4.8
Cold anthropomorphic	Anger	−*	−*		−*	−0.4	57.4		22.0	−6.0	5.5
	Ring CZT	−*	−*		−*	N/A	54.0		17.3	N/A	4.8

^*^The anthropomorphic phantom was not imaged with ^99m^Tc

### ^177^Lu measurements

#### NEMA phantom

**Fig. 4:**
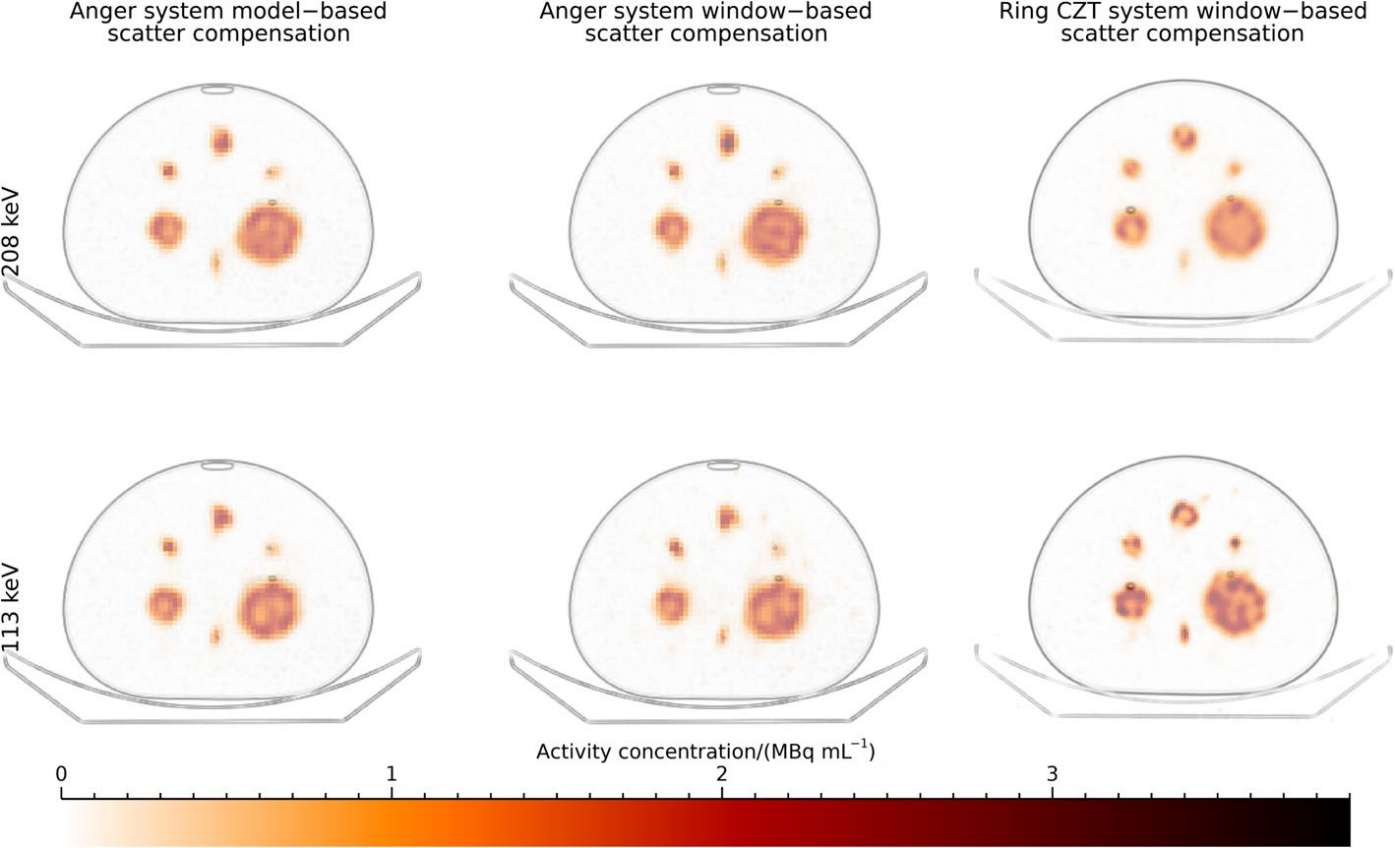
Examples of reconstructed images for the NEMA phantom filled with.^177^Lu. Images are reconstructed with 10 iterations (10 subsets)

Examples of transaxial images of the NEMA phantom filled with ^177^Lu, reconstructed with 10 iterations and 10 subsets are shown in Fig. 4. The images acquired at 208 keV are visually similar for the two systems, whilst for the image acquired at 113 keV the image from the ring-configured CZT system appear sharper.

**Fig. 5:**
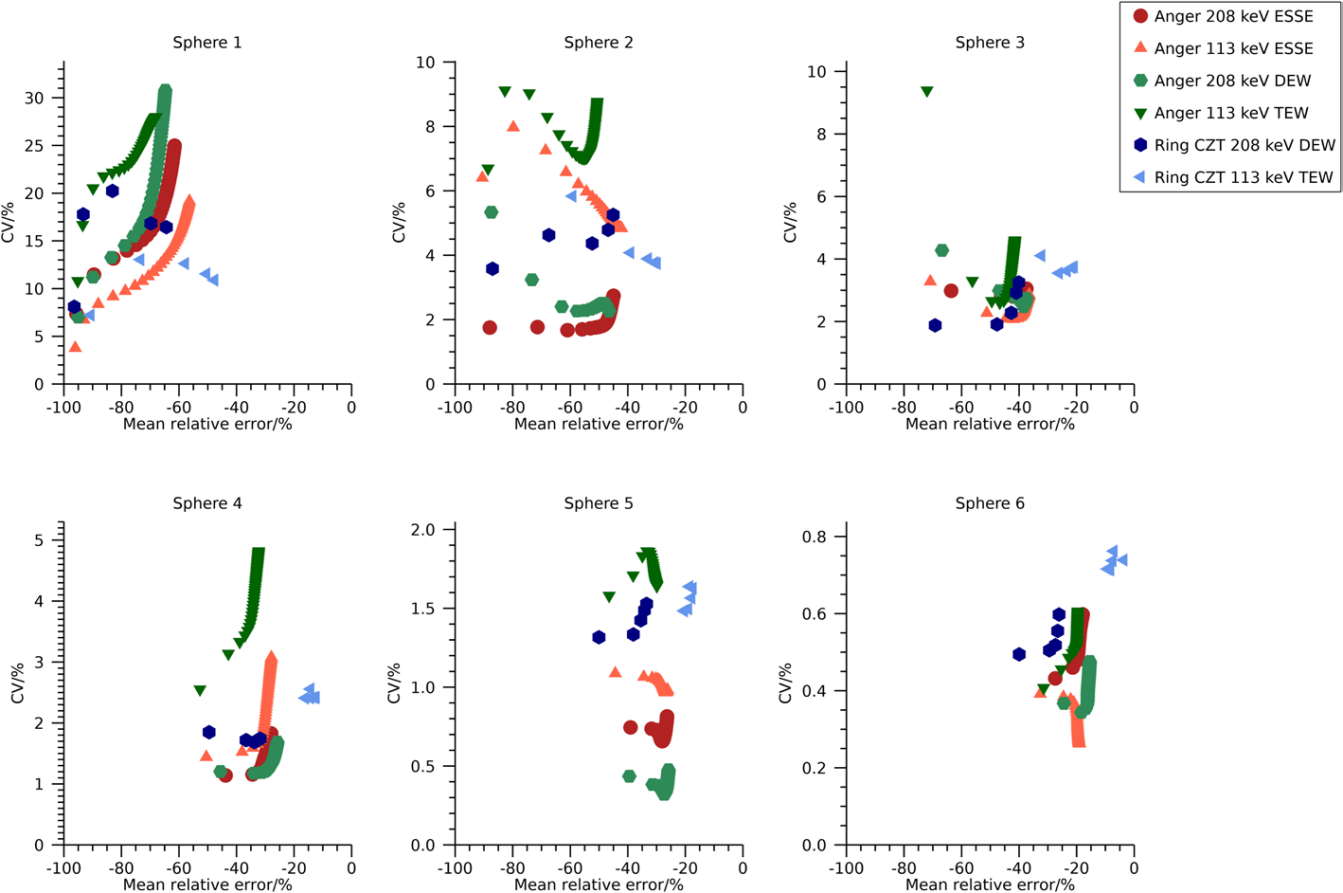
Coefficient of variation versus mean relative error for the NEMA phantom filled with ^177^Lu. Each point represents a specific number of iterations. Note that the ordinate is individual for each sub-plot

Plots of mean relative error versus CV for the different reconstructions of the NEMA spheres are shown in Fig. 5 and numeric data at 30 iterations are presented in Table 3. The two camera systems result in similar mean relative errors and CVs when using the 208 keV energy window. For the Anger system, ESSE and window-based scatter compensation result in similar values. For the 113 keV energy window, the ring-configured system results in lower mean relative errors for similar CVs as the Anger system, which is in line with the visual differences observed in Fig. 4.

**Fig. 6:**
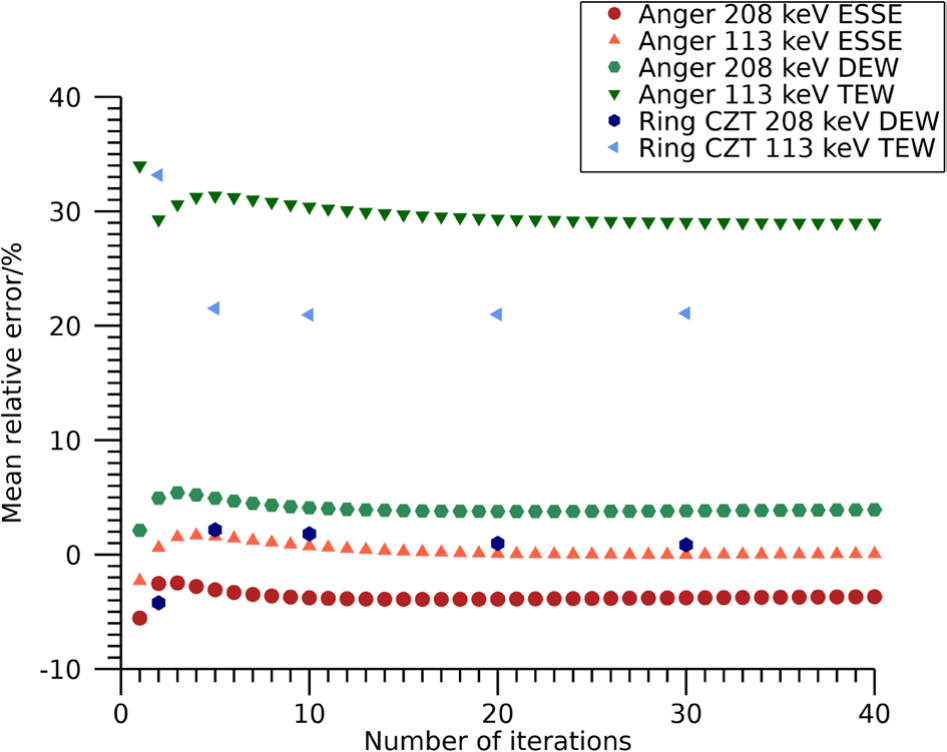
Mean relative error in estimated total activity in the NEMA phantom filled with ^177^Lu. Results are presented as function of number of iterations. The number of updates is ten times the number of iterations

Mean relative errors in estimated total activity in the phantom are shown in Fig. 6 as function of number of iterations. The total estimated activity stabilizes after a few iterations and differ substantially between energy windows and reconstruction methods. The activities from the 113 keV energy window for the ring-configured CZT system and the Anger system when using TEW are overestimated by more than 20%, whilst the other windows and scatter compensation methods result in mean relative errors below 10%. Numeric data at 30 iterations are presented in Table 4.

#### Anthropomorphic phantom

**Fig. 7:**
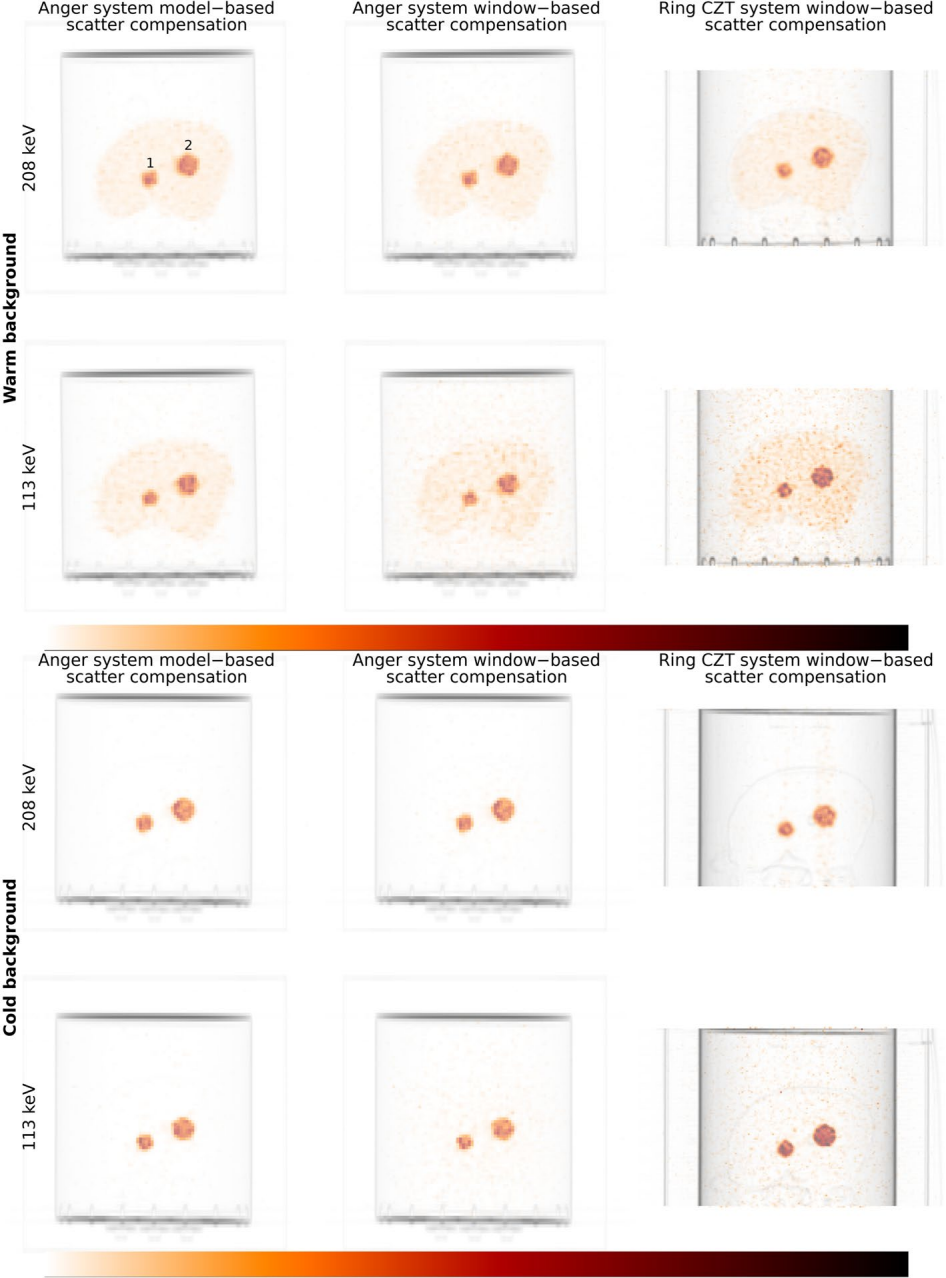
Examples of maximum-intensity projections of SPECT images for the anthropomorphic phantom. Images are reconstructed with 10 iterations (10 subsets). Sphere positions are indicated for the images with warm background from the Anger system using ESSE

Examples of maximum-intensity projections of the anthropomorphic phantom with warm and cold background reconstructed with 10 iterations and 10 subsets are shown in Fig. 7. For the ring-configured system, signal is clearly seen outside the spheres and liver for the imaged with cold and warm background, respectively. There are visible streaks in the axial direction for the 208 keV energy window from the ring-configured CZT-system for the phantom with cold background.

**Fig. 8:**
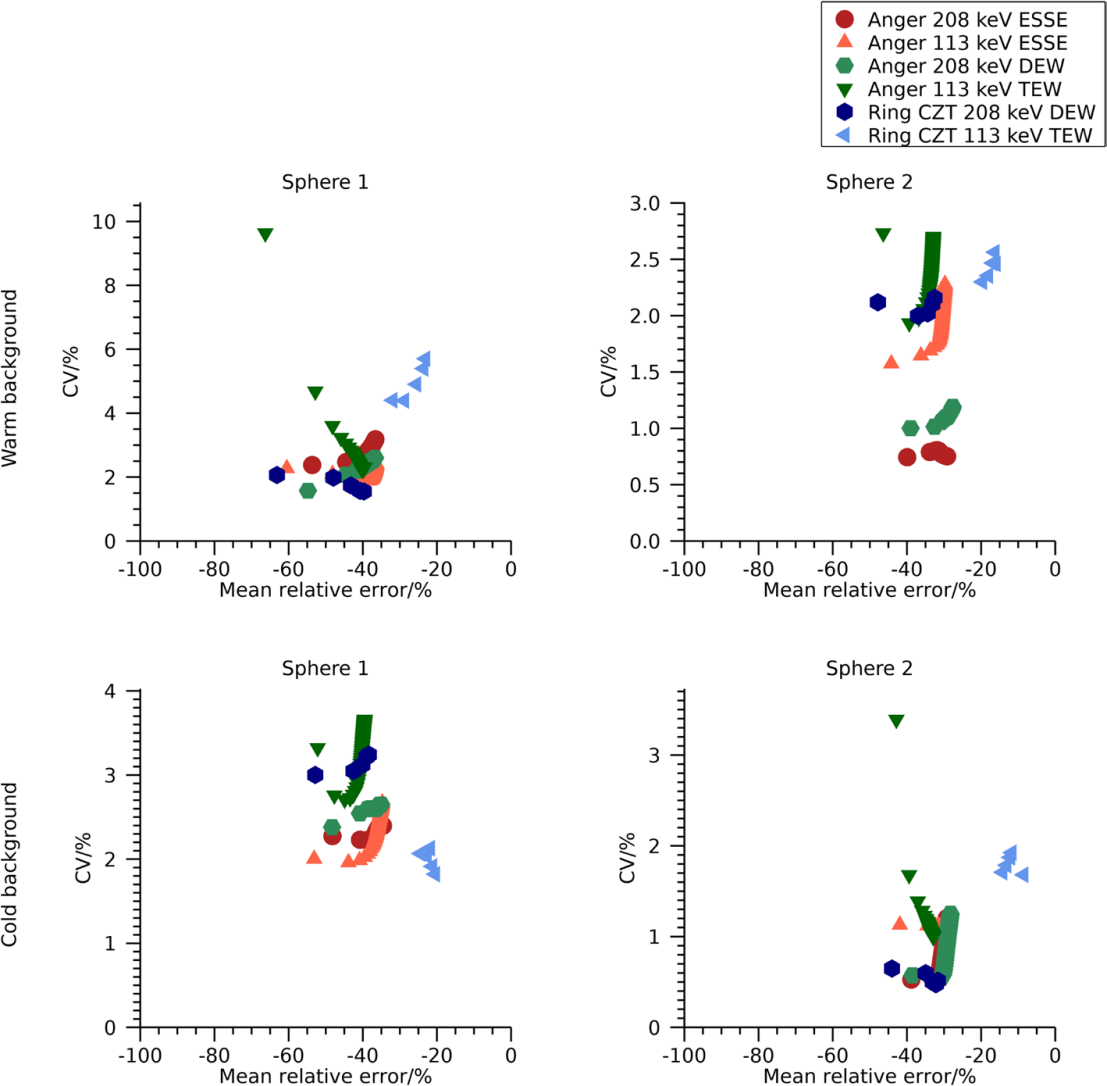
Coefficient of variation versus mean relative error for the anthropomorphic phantom. Note that the ordinate is individual for each sub-plot

Plots of mean relative error versus CV for the different reconstructions with cold and warm background are shown in Fig. 8 and numeric data at 30 iterations are presented in Table 3. Differences between systems and scatter-compensation methods are similar as for the NEMA phantom, with the main difference between systems being the lower bias for the ring-configured CZT system when using the 113 keV energy window.

**Fig. 9:**
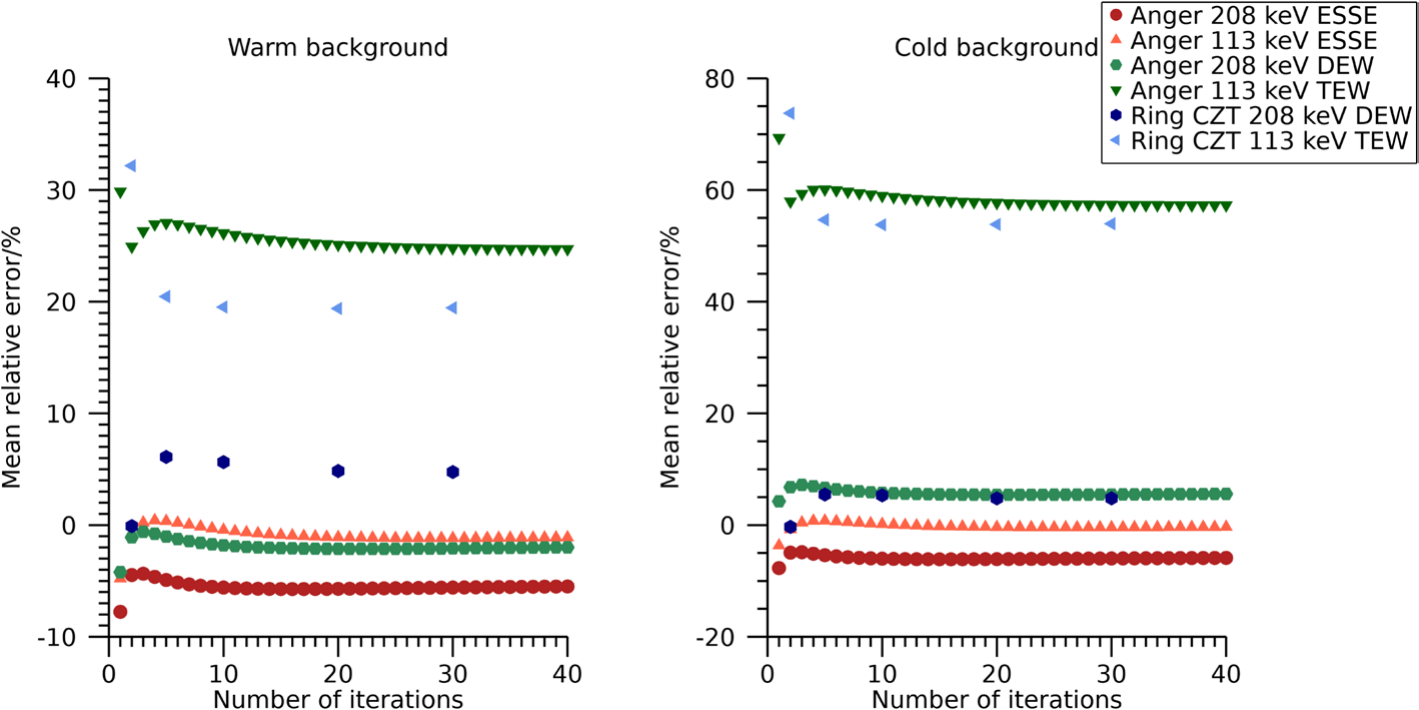
Mean relative error in estimated total activity in the anthropomorphic phantom. Results are presented as function of number of iterations. The number of updates is ten times the number of iterations. Note that the ordinate is individual for each sub-plot

Mean relative errors in estimated total activity for the anthropomorphic-phantom measurements are shown in Fig. 9. Numeric data at 30 iterations are presented in Table 4. Similar trends are seen as for the NEMA phantom.

#### Estimated total activity for extended acquisition time

**Fig. 10:**
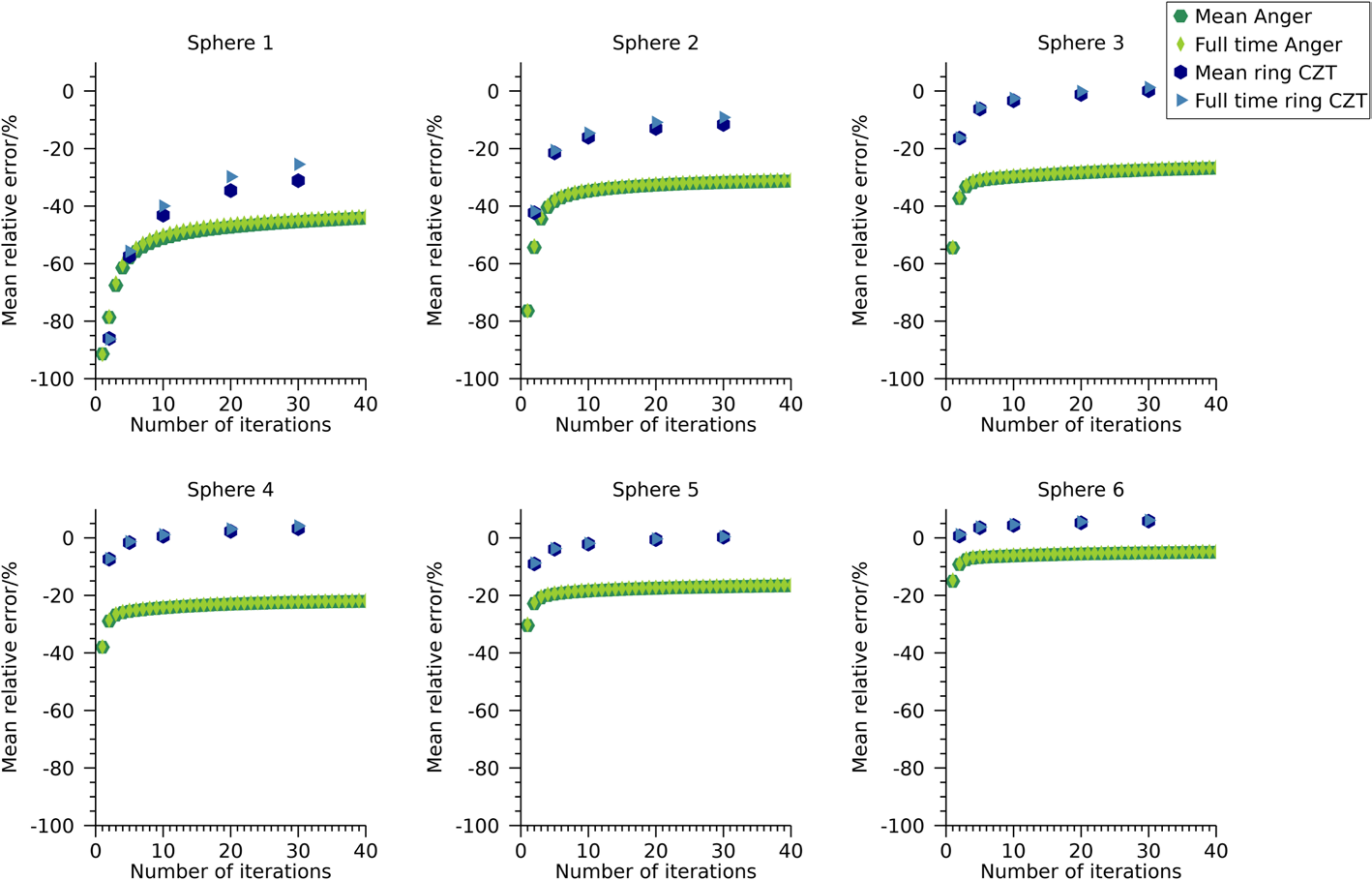
Mean relative error for ^99m^Tc activity concentration in the NEMA phantom for different acquisition times

**Fig. 11:**
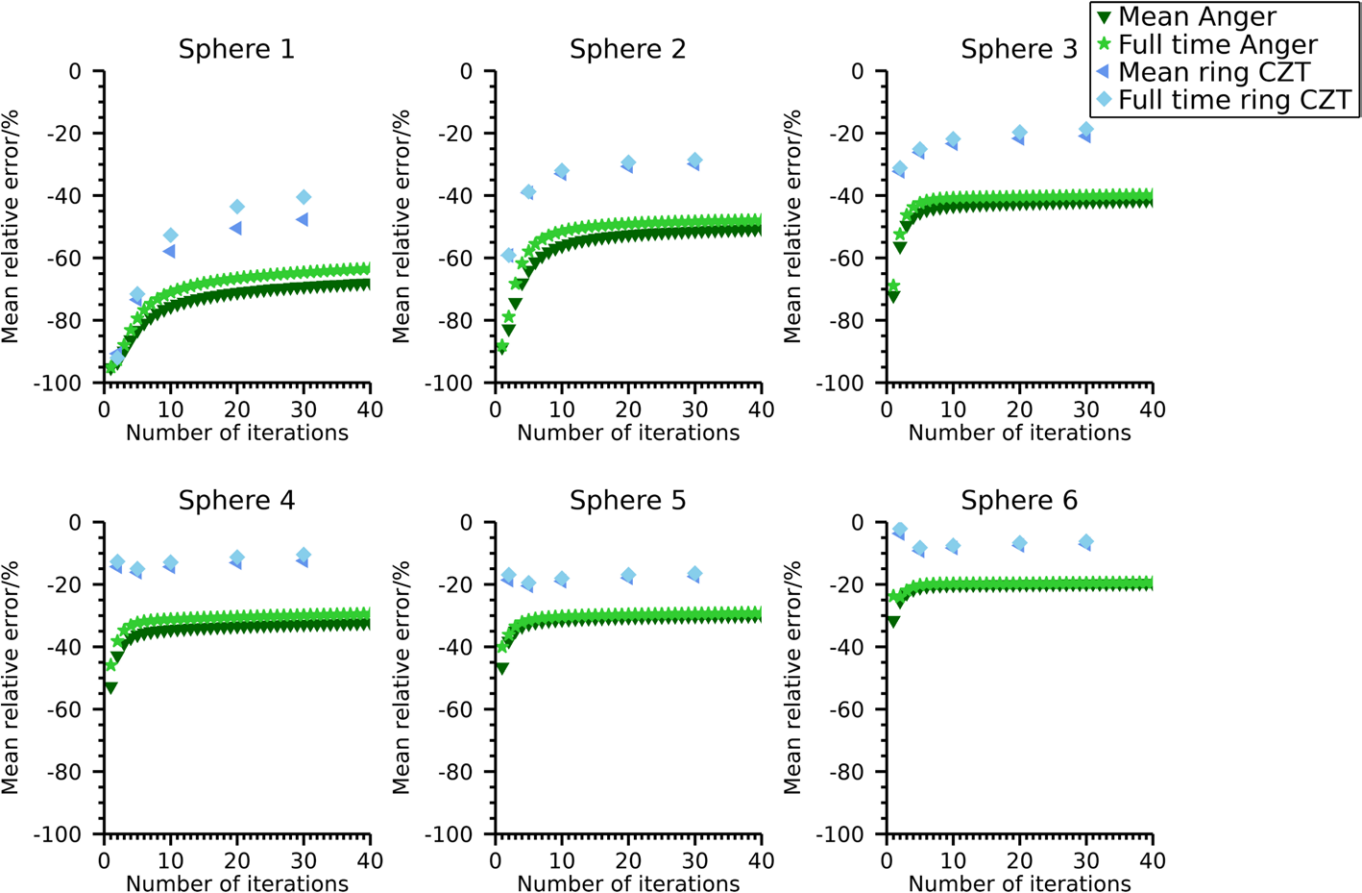
Mean relative error for ^177^Lu activity concentration in the NEMA phantom for different acquisition times. Estimates are for an energy window around 113 keV

**Fig. 12:**
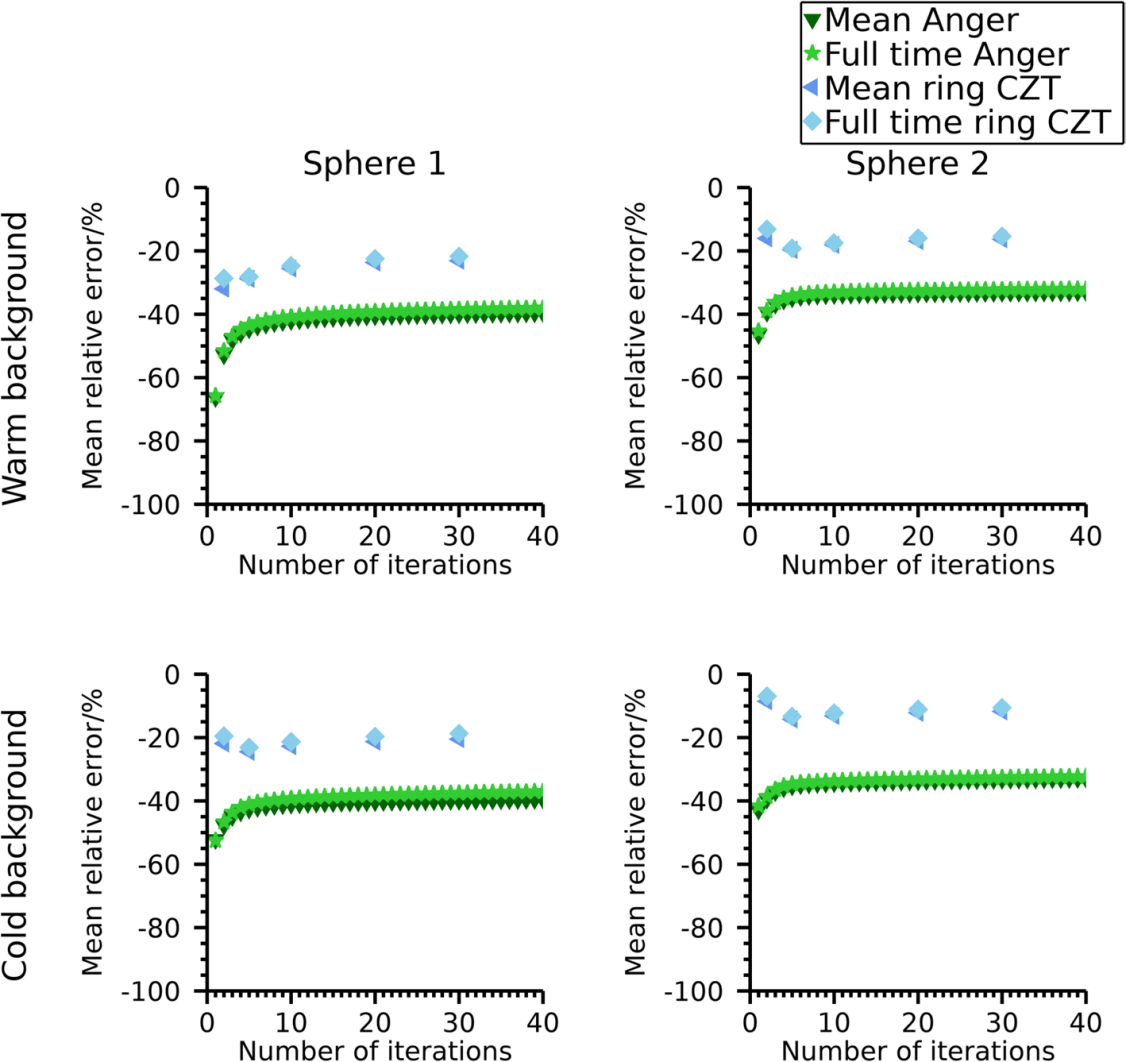
Mean relative error for activity concentration in the anthropomorphic phantom for different acquisition times. Estimates are for an energy window around 113 keV

To further elaborate on the overestimation of activity observed for the window-based scatter-compensation methods for ^99m^Tc and ^177^Lu at 113 keV, images were also reconstructed using the full-time data for these cases. The relative errors in total activity in the phantoms at 30 iterations are presented in Table 4. The relative activity-concentration error as function of number of iterations are presented in Fig. 10, Fig. 11, and Fig. 12 for the NEMA phantom filled with ^99m^Tc, the NEMA phantom filled with ^177^Lu, and for the anthropomorphic phantom, respectively. The mean relative errors over timeframes are also indicated for reference. The relative errors are reduced compared with the mean error over timeframes, while the errors in activity concentrations remain relatively unaffected.

## Discussion

There is currently an interest for exploring new gamma-camera designs for RNT dosimetry [12–15]. These systems have claimed potential advantages in terms of, *e.g.*, better energy resolution, better spatial resolution, and better system sensitivity, but their use for RNT dosimetry requires them to be used outside their normal field-of-application in diagnostics. Hence, there is a need for better understanding of the properties of activity-concentration estimates from these systems and their potential advantage compared with conventional systems. This paper contributes to this effort by evaluating bias and precision for ^177^Lu activity-concentration estimates from 113 and 208 keV energy windows using a ring-configured CZT SPECT system in comparison with a dual-headed Anger camera. Furthermore, corresponding properties were studied for ^99m^Tc since this is the most commonly used radionuclide for SPECT imaging and thereby is relevant as a baseline to which results for ^177^Lu can be related.

A problem when comparing bias and precision between systems is the inherent conflict between these two properties in modern tomographic-reconstruction methods, where, in practice, almost any noise level can be achieved just by using a sufficiently high number of updates. Hence, noise and bias for a specific number of iterations is not particularly relevant as measures of system performance, and these parameters have to be studied in combination [12, 30, 31]. When evaluating noise properties of new SPECT systems, common strategies have been to consider the voxel-to-voxel variability within a VOI representing a uniform region in the phantom or as the variability of concentration estimates within targets or background [12–15, 22]. These kinds of noise measures are often relevant for evaluation of diagnostic tasks but are of limited relevance for RNT dosimetry where individual voxel values or variability in background are seldom of interest [32, 33].

A more relevant noise-measure is the repeatability of estimates over repeated image acquisitions, which was enabled by the use of long acquisitions with subsequent partitioning into independent time frames from list-mode data in the current study. The evaluation of noise on the region level rather than on the voxel level also had the advantage that the effect of the different voxel sizes of the images from the Anger system and the ring-configured CZT system was diminished. However, as only a small number of time frames (six) was available, the CV estimates are in themselves relatively uncertain [34] and sensitive to outliers. We believe this is also the main reason why some of the CV versus mean relative error do not follow the expected pattern, *i.e.*, that the CV increases as the bias decreases.

Our data suggest a similar performance of the ring-configured CZT system and the conventional dual-headed Anger system for the investigated evaluation metrices when using an energy window around 208 keV for ^177^Lu, but a possibly better performance of ring-configured system when using data for an energy window around 113 keV and for ^99m^Tc. In particular, the ring-configured system demonstrated a considerably lower bias than the Anger system. It could be noted that the Anger system studied was for a 5/8″ crystal, which typically deteriorate the intrinsic spatial resolution but improves sensitivity compared with a standard system with 3/8″ crystal. Since the system spatial resolution is dominated by collimator effects, the effect on bias should be small, while, specifically for 208 keV, the thicker crystal results in a higher sensitivity and thus, potentially, a reduced CV for the Anger system.

Unfortunately, the use of window-based scatter compensation methods led to relatively large errors in total activity in the phantom for ^99m^Tc and ^177^Lu when using the energy window around 113 keV. This is a problem for both the Anger system and the ring-configured CZT system. This is a complication for the interpretation of the differences in recovery between systems since a reduced negative bias in estimated activity concentration could also result from a general overestimation of activity rather than an improved spatial resolution. The reason for the overestimation is indicated in Fig. 7, where signal is present also outside the liver for the 113 keV images reconstructed with TEW, in line with the overestimated activity being the result of poor scatter compensation.

Window-based scatter compensation is known to have limited accuracy [35, 36] and there is an inherent conflict between the need for scatter windows to be representative of the scatter in the main window and the need for high signal-to-noise ratios of scatter estimates. It is possible that further optimization of energy-window settings could improve the performance of these methods for the estimation of total activity, but this was not pursued in the current study. For the ring-configured CZT system, it is likely that the performance would also improve by using modified versions of the DEW and TEW methods to account for the contamination of scatter windows by primary events due to charge-sharing [20, 21]. At the time-of-writing, such modified DEW scatter correction is implemented for imaging of ^177^Lu at 208 keV at the StarGuide system but not for any other radionuclides or energy windows. This is a potential reason for the relatively good performance of this system at 208 keV with respect to the estimation of total activity in the phantoms.

The SPECT images with longer acquisition time demonstrated a better preservation of the total activity (Table 4), while the activity concentrations in the spheres were relatively unaffected (Table 3). Hence, the low biases of the activity-concentration estimates from the 113 keV energy window for the ring-configured system did not seem to be the result of a general overestimation of activity. Since the gross overestimation of total activity is a phenomenon that is not observed when using model-based scatter compensation for the Anger system, there is reason to believe that the problem would diminish also for the ring CZT system if such methods were implemented. However, such methods are also associated with issues not studied in the current project, *e.g*., the effect of scatter from activity outside the image field-of-view, that window-based methods do not suffer from. The potential negative consequences of overestimating the total activity would tend to be application dependent. The consequence for, *e.g.*, renal dosimetry or tumour dosimetry would be expected to be small as long as the activity concentration in the target itself is accurate, whilst the consequences for, *e.g.*, bone-marrow dosimetry would be more severe due to the need to estimate low activity concentrations and the higher importance of cross-dose for this organ. Since one of the main reasons for the interest in ring-configured CZT systems for ^177^Lu activity estimation is the potential to shorten acquisition times [17], we believe that the accuracy of scatter compensation and the preservation of total activity are important to consider when evaluating the performance of the system.

One limitation of this study is the stylized geometries used. The NEMA PET body phantom has emerged as the de-facto standard for evaluating quantitative performance for SPECT imaging but is limited in terms of realism. The modified version with the smallest sphere replaced with a large sphere with a diameter of 6 cm is an attempt to make the geometry more relevant for SPECT imaging, but the geometry is still stylized. However, these geometries with spheres of gradually increasing volume is also valuable since it allows for a systematic evaluation under well-defined conditions. Hence, we believe these experiments are important when evaluating these kinds of new systems and comparing them with standard equipment. Furthermore, the anthropomorphic phantom employed, whilst still stylized, is a step towards increased realism and showed results similar to the NEMA phantom.

Recent years have seen the introduction of maximum á posteriori (MAP) reconstruction methods [37, 38] into clinical SPECT imaging, which is partly correlated to the introduction of ring-configured SPECT systems. These methods are designed to avoid the deteriorating signal-to-noise ratio inherent to the OS-EM algorithm as the number of updates increases and have generally been deemed favourable for a number of tasks [39–41], although yet mainly for PET applications. The StarGuide system implements the BSREM method [37], but we chose not to utilize this capability for the current study. The reason for this was partly that at a first instant we wanted to evaluate the effect of different hardware configurations, and thus we preferred to use the de-facto standard algorithm for SPECT reconstruction (*i.e.*, OS-EM), and partly because, in our experience, matching parameters between different implementations of MAP is difficult. Hence, designing a fair comparison between the ring-configured CZT and dual-headed Anger system would be challenging if also involving these more advanced reconstruction methods. However, in light of the potential advantages of MAP reconstruction methods, we believe this is an issue where further research is of interest.

The comparable CVs for activity-concentration estimates from the two systems considered indicate that the potential for using ring-configured gamma-cameras to shorten acquisition times in ^177^Lu-SPECT imaging is limited compared with standard systems. Rather, we would argue that the potential for substantially shortening acquisition times is already present for standard systems [42], provided that the aim is to quantify mean activity concentration in reasonably large regions. The possibility to use focused acquisition is one way to locally increase signal-to-noise ratios for ring-configured systems that has been explored in a number of studies [9, 22, 23] and that is not possible using dual headed systems with parallel hole collimators. However, this feature would, in our opinion, be difficult to use for typical ^177^Lu dosimetry applications, where it is often desirable to acquire information about risk organs (*e.g.*, left and right kidneys) as well as tumours.

The improved recovery for ^177^Lu when using the 113 keV energy window is encouraging for the use of ring-configured CZT systems for clinical dosimetry, although the errors observed in total activity are of potential concern. Furthermore, the performance when using the 208 keV window seems to be similar as for the dual-headed Anger system. However, penetration seems to be a problem for this energy, as visually demonstrated by the axial streaks in the maximum-intensity projections in Fig. 7, with similar effects previously noted by Danieli et al. [12]. As such, one important aspect to note is that the reconstructions performed for the CZT system included compensation for penetration, which we believe is one of the main reasons for the good performance of this camera despite the collimator not being primarily designed for imaging at these energies. This also raises the question if similar results could be obtained for the Anger system if equipped with similar collimators and including full collimator-response modelling in the reconstruction projection-formation model for this higher energy. There is reason to believe that the performance of both systems could be improved provided that collimators were specifically optimized for quantitative imaging of ^177^Lu.

## Conclusions

Ring-configured CZT gamma-camera systems are viable alternatives to dual-headed Anger systems for SPECT-based activity-concentration estimation of ^177^Lu. The GE StarGuide system has a potential advantage for estimation of activity concentration compared with the GE Discovery 670 system with medium-energy collimators for imaging using an energy window around 113 keV with lower bias for similar precision, whilst performance is similar for imaging using the 208 keV photons. However, the preservation of total activity is a potential concern and a factor that must be considered.

## Data Availability

Data are available from the corresponding author on reasonable request.

## Abbreviations

BSREM: Block-sequential regularized expectation maximization
CV: Coefficient of variation
CZT: Cadmium-zinc-telluride
DEW: Dual-energy window
ESSE: Effective source scatter estimation
FOV: Field-of-view
LEHR: Low-energy high-resolution
MAP: Maximum á posteriori
MEGP: Medium-energy general-purpose
OS-EM: Ordered subsets expectation maximization
PET: Positron emission tomography
RNT: Radionuclide therapy
SPECT: Single photon emission computed tomography
TEW: Tripple-energy window
VOI: Volume of interest
